# Costs of integrating demand-based reproductive health commodity model into the Government and NGO service delivery systems in Bangladesh: a supply side perspective

**DOI:** 10.1186/s40064-015-1610-6

**Published:** 2015-12-23

**Authors:** Ziaul Islam, Abdur Razzaque Sarker, Shahela Anwar, Humayun Kabir, Rukhsana Gazi

**Affiliations:** Health Economics and Financing Research Group, Centre for Equity and Health Systems, International Centre for Diarrhoeal Disease Research, Bangladesh (icddr,b), Dhaka, Bangladesh

**Keywords:** Cost, Reproductive health, Service delivery

## Abstract

To estimate additional total cost and average cost of integrating the demand-based reproductive health commodity model into the existing Government and NGO facilities in Bangladesh. Activity based cost analysis was conducted during 2006–2008 in two low performing rural sub-districts (*Nabigong and Raipur sub*-*district*) and one urban slum area in Dhaka city, Bangladesh. Activity-based cost data were collected using ingredient approach, which comprised of listing all types of inputs by activity, quantities and prices for each input. Total cost was presented according to capital and recurrent items. The supply side perspective was considered for entire analysis. The total cost of integrating demand-based reproductive health commodity (DBRHC) model into the Government and NGO service delivery system was estimated to BDT 18,667,634 (US$274,524). The proportion of capital cost was 59 % and the recurrent cost was 41 % of the total cost. The average cost per beneficiaries was BDT 230 (US$3.38) only for introducing this model into the existing health system. The built-in interventions of DBRHC model were doable at low-cost at the selected Government and NGO settings at the grass-root level. The model has potential of further cost containment during scaling up—if the intervention costs are adjusted with the existing functionaries of the Government and NGOs.

## Background

For achieving universal coverage in reproductive health and family planning services, access to user-friendly reproductive health commodity is essential (UNFPA 2007). Client’s individual choice and preferences particularly for meeting the unmet reproductive health need is instrumental in improving such coverage. Back in 1994, a demand-based strategy focused more on client’s choice and preference was advocated in the Plan of Action of the international Conference on Population and Development (ICPD-5 1994). It is encouraging to see that Bangladesh has made remarkable progress towards achieving the MDG 4 and 5 specifically in the progressive reduction of maternal mortality ratio from 320 in 2001 to 194 in 2011 per 100,000 live birth, under five mortality ratio from 146 in 1991 to 53 in 2011 per 1000 live birth and total fertility rate (TFR) from 6.5 in 1970 to 2.3 in 2011 (BDHS 2013; UNDP 2013). Alongside Government of Bangladesh (GoB), NGOs and other development partners have been playing a significant role in this endeavor (Balabanova et al. 2011). However, the country is still facing challenges in improving the utilization of family planning and reproductive health services at the community level. One of the challenges is to introduce a demand-based service delivery system as opposed to conventional supply-driven family planning and reproductive health care services. A recent analysis of data from the Bangladesh Demographic and Health Survey (BDHS) provides clear evidence of gap between the rich and poor in a range of health and population indicators, including fertility and use of family planning and other reproductive health services (BDHS 2013). Poorer women have more children compared to well-off women, with a TFR of 3.1 among the poorest income quintile and 1.9 among the richest quintile respectively (BDHS 2013). The TFR varies widely across different geographic areas of Bangladesh; for instance, its’ quite high in Sylhet (3.1) and Chittagong division (2.8) compared to TFR for the national level (2.3) (BDHS 2013). It is also notable that there are intra-urban differentials in reproductive health and family planning indicators between slum and non-slum populations. In Bangladesh, the average age of first pregnancy is 17 years and about 35 % of adolescent girls are either pregnant or already have a child (BDHS 2009).These low-performing areas and vulnerable populations need special interventions.

In the backdrop of the aforesaid scenario, the National Institute of Population Research and Training (NIPORT) under the Ministry of Health and Family Welfare (MoHFW) lunched a Demand-based Reproductive Health Commodity Project in 2005. Duration of the project was for 3 years. The goal of this project was to modify the existing reproductive health service delivery system of the government and partner NGOs in selected vulnerable areas to improve the efficiency and quality of service delivery with engagement of the community. In the project period, the entire chain of reproductive and family planning service provision and community awareness was improved through developing and testing a series of interventions, addressing the demand- and supply-side issues with particular emphasis on demand side perspectives. The project was implemented in collaboration with the Population Council Bangladesh, RTM international, JSI Deliver and icddr,b under technical and financial assistance of United Nations Population Fund (UNFPA) and Canadian International Development Agency (CIDA).

This paper looks into the additional input costs of integrating the above-mentioned demand-based reproductive health commodity model into the existing Government and partner NGO facilities in Bangladesh. Specifically it estimates the average cost and total additional cost of implementing the model by interventions indicating the cost implications of scaling up into the Government and NGO service delivery system using their existing functionaries.

## Demand-based reproductive health commodity model

**Fig. 1:**
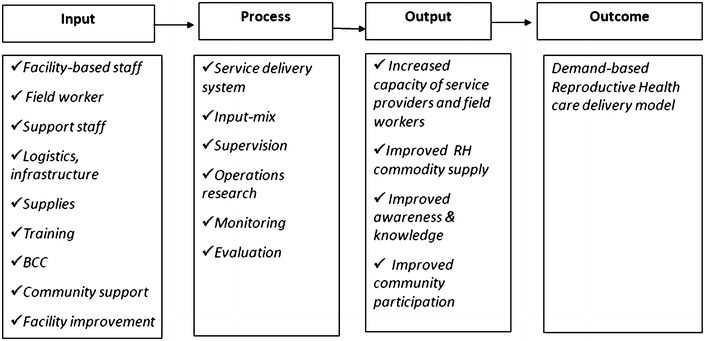
Conceptual framework of demand-based reproductive health commodity model

Based on an initial needs assessment of the project, it was determined that capacity development of service providers and their supervisors on reproductive health issues, related Behavior Change Communication (BCC) interventions, community activation, improvement of physical condition of facilities and strengthening supply system of the selected facilities are the key areas that need to be addressed to improve the efficiency of low performing areas and quality of service delivery. Based on the findings of the needs assessment, the demand-based reproductive health commodity (DBRHC) model was designed and implemented with four different cross-cutting interventions. The first three interventions were designed for rural setting and the fourth one was designed for urban setting in Bangladesh. Although the specific aims of these interventions were different from each other, they all were complementary and supplementary to each other. The first aim was to increase the use of modern contraceptive methods and decrease the discontinuation rate during reproductive life cycle. Secondly, to create service space for men and youth at Health and Family Welfare Centres (HFWC) and raise knowledge of unmarried males on reproductive health including sexually transmitted infections (STI) and reproductive tract infections (RTI) as well as to increase utilization of reproductive health services by male and youth from HFWCs. Thirdly, to develop a system of voucher distribution among the poor pregnant women for quality maternal health services including ANC, PNC and delivery, and increase the capacity of service providers for offering quality maternal health care. The fourth aim was to improve the capacity of NGO service providers, strengthen service delivery points and improve the counseling services in the urban areas. For meeting the above objectives a number of initiatives were undertaken such as, capacity development of service providers, improvement of physical infrastructure and supply system of health facilities, creating awareness of family planning and reproductive health issue through BCC activities among eligible couples, establishment of peer promoters, arranging waiting space for men and youths, altering service delivery to make it more accommodative to men, display board at the waiting area, ensured privacy and adequate RH commodity supply at the facilities, designing a voucher distributing system, strengthening service delivery points, improving counseling services. The framework of this model is described below in Fig. 1.

An evaluation of the project was conducted by icddr, b that comprised of comparison between baseline and end line data to examine the effectiveness of the project. Evaluation findings showed that all four interventions were very effective in achieving improved reproductive health outcomes (Gazi et al. 2013, 2014 ; Kabir et al. 2013, 2014).

## Methods

### Study site and population

The project sites were located in low performing rural and urban areas of Bangladesh. The rural sites were chosen among the low performing public facilities at *Nabigonjupazila* (sub-district) of Hobigonj district and *Raipur upazila* (sub-district) of Lakshmipur district under Sylhet and Chittagong division respectively (Kabir et al. 2013). The urban sites were selected from the slums of Dhaka city having a partner NGO-program. The study was implemented during 2006–2008. A total of 81,183 persons were considered as the beneficiaries of the project during the entire period.

### Study perspective

For calculating costs of integrating this model into the service delivery system of the selected facilities, provider’s perspective was considered. This cost calculation covered only the additional costs of field activities related to the implementation of the model.

### Measuring cost

Activity-based cost data were collected using ingredient approach, which comprised of listing all types of inputs by activity, quantities and prices for each input (Drummond et al. 2005). The total cost data included a comprehensive list of capital and recurrent cost items (Table 1). Total cost was divided by the total number of potential beneficiaries for estimating provider’s average cost per beneficiary. Costs incurred upon the project for paying salary of its research investigators for pre- and post-intervention surveys, baseline and end line surveys and monitoring-evaluation have been excluded from this calculation. It is not expected that the government or partner NGOs would spend their resources for hiring such staff at higher rate or undertake large surveys for testing similar intervention as the project did.

**Table 1:** Total cost, share of unit cost and cost per beneficiary, BDT (USD)

Cost category	Parameters	Total cost	Unit cost	% of unit cost
Capital cost	Basic training	2,834,434 (41,683)	34.91 (0.51)	15.18
	Workshop	1,104,207 (16,238)	13.60 (0.20)	5.92
	BCC activities	3,046,459 (44,801)	37.53 (0.55)	16.32
	BCC materials	3,497,560 (51,435)	43.08 (0.63)	18.74
	Facility improvement	501,633 (7377)	6.18 (0.09)	2.69
Sub-total		10,984,293 (161,534)	135.30 (1.99)	58.84
Recurrent cost	Stationeries and office supplies	1,402,467 (20,625)	17.28 (0.25)	7.51
	Refresher training	487,912 (7175)	6.01 (0.09)	2.61
	Meeting-orientation, coordination	2,324,713 (34,187)	28.64 (0.42)	12.45
	Cell phone bill, utilities	83,306 (1225)	1.03 (0.02)	0.45
	Local travel	2,902,337 (42,681)	35.75 (0.53)	15.55
	Voucher scheme	482,606 (7097)	5.94 (0.09)	2.59
Sub-total		7,683,341 (112,990)	94.64 (1.39)	41.16
Grand total		18,667,634 (274,524)	229.95 (3.38)	100.00

### Capital costs

Capital items or inputs are defined as goods that last more than 1 year and typically have a unit cost greater than US$100 (Creese and Parker 1994). However, capital costs or assets are usually invested at a bulk amount and used over time (Drummond et al. 2005). Capital inputs in the project included non-recurrent basic training and workshop with target groups, behavior change communication (BCC) activities (e.g., drama, video show, cultural events, and observance of world population day/national days), BCC material development and printing (e.g., flip chart, brochure, signboard, board making, booklet, guidelines) and physical improvement of selected facilities (e.g., power line set-up, furniture and curtain supply, building repair and painting). For calculating annualized values of capital items, useful life time of inputs and 5 % discounting rate was considered (Drummond et al. 2005). Economic value of each of these capital inputs was then calculated for the total project period of 36 months (2006–2008).

### Recurrent costs

Costs associated with inputs that were consumed/used-up in the course of a year and procured regularly were considered recurrent costs. These input costs included refresher training, local travel, stationeries and office supplies, bills for phone calls and utilities, cost of coordination meeting and printing, and allowances paid for maternal voucher scheme during 36 months of the study. Refresher training cost was included for staff directly involved in service delivery.

### Data sources

Necessary cost data were gathered from the project databases and key-informant interviews of the two partner organizations (i.e., RTM International and Population Council, Bangladesh).

### Data analysis

Data were entered into Microsoft Excel 2007. All entries were manually double-checked and verified. Data were presented as average cost, total cost and share of total cost in local currency, i.e., Bangladeshi Taka (BDT) and US dollars (US$) applying the exchange rate (US$1 = BDT 68) during the mid-point of the data collection year (2008).

## Results

The total cost of integrating DBRHC model into the government and NGO service delivery system was estimated to BDT 18,667,634 (US$274,524). The proportion of capital cost was approximately 59 % and the recurrent cost was 41 %. Among capital cost, BCC materials was the largest cost-driver, followed by BCC activities (Table 1). Local travel comprised the highest cost among all recurrent cost components. Considering the total number of beneficiaries (N = 81,183), the average cost was BDT 230 (US$3.38) for introducing this model into the existing health system.

## Discussion

In Bangladesh, the government and NGOs have been maintaining reproductive health service delivery systems in urban and rural areas that are largely supply-driven. Evidences show that the systems suffer from various programmatic shortcomings and lack of adequate community engagement resulting in poor quality of care and low utilization (Oxford 2005). The DBRHC model actually introduced some additional programmatic elements into the current systems to address those shortcomings and produced better outcomes (Gazi et al. 2009). This cost estimation was targeted to capture the costs of additional inputs introduced at the field level only during the period of implementation. Making the cost information useful for the end users and policy maker including managerial level, the study addressed the cost per beneficiary and also the complete cost of the DBRHC model. With costs presented in this categorical way, the manager as well as health care provider can better consider additional cost of adding individual component to the existing healthcare delivery system. This cost analysis also makes a comparisons between existing healthcare programs and will help to project the future cost of the program that are largely affected by the number of beneficiaries.

In the analysis, we found that for introducing this model into the existing health care system of Bangladesh, a total of BDT 18,667,634 (US$274,524) is needed where the capital cost incurred the main share. Of total cost components, BCC related materials and activities appeared as the main cost-drivers of the model. Among the recurrent cost local travel cost was the highest followed by the cost of meeting, orientation sessions and coordination meeting and meeting with members of the community support groups. Voucher scheme was the smallest cost component of the model since over the entire project period only 580 pregnant women were enrolled in this scheme (Table 2).

**Table 2:** Potential beneficiaries of DBRHC model

Component/interventions	Number of beneficiary of DBRHC model
Creating space for men and youth at H and FWC	28,996
Rational use of modern contraceptive methods for married woman	21,050
Improving the quality of FP and RTI services for married Women of reproductive age	30,557
Voucher scheme for pregnant woman	580
Total	81,183

We found that for incorporating the model into the existing healthcare system the average cost per beneficiary was BDT 230 (US$3.38). It has been found in another study that MNCH services including BCC for poor people in the urban slums of Bangladesh incurred a total cost of BDT 1332.8 or US$19.25 per beneficiary, where the average cost for normal delivery was BDT 1167 or US$17.0 (Islam et al. 2010) and BCC related activities was 165.8 or US$2.25 (Sarker et al. 2013). It implies that adding up demand-based reproductive health services into the existing service facilities can reduce cost instead of developing new facilities and or services. However, this study did not consider the demand side perspectives, although free maternity services in Bangladesh impose large out of pocket expenditures on patients (Khan 2005). From an earlier study it was found that higher medical cost was the main concern that impeded clients from seeking care for complications during pregnancy (Koenig et al. 2007). The poorer women faced greater challenges in receiving the treatment as their families/relatives were not able to arrange necessary cash (Pitchforth et al. 2006). Hence it is not equitable in terms of receiving quality of care. Further, human resources for health and inequity of its distribution are another area of concern towards developing an effective, efficient and equitable health system (Ahmed et al. 2011). This DBRHC model actually introduced some additional programmatic elements into the current healthcare system to address the poor quality of care and low utilization and produced better outcomes. However the present study has some limitations as the cost of household for receiving treatment or opportunity costs were not considered. Further, the study did not consider any other client related out of pocket expenses.

The interventions under DBRHC and related cost information indicate that if scaled up by the Government, it has scope of further cost containment through cost adjustment; particularly with respect to BCC material inputs (material development and printing)—because the Ministry of Health and Family Welfare has its own well-equipped BCC unit/Health Education Bureau and printing unit under its Directorate General of Health Services and Directorate General of Family Planning that could produce similar materials at lower cost. In addition to its in-house arrangement; the MoHFW has collaborative linkage with the Department of Mass Communication for BCC campaign under the Ministry of Information and the Bangladesh Government Press (BG press) for printing, publication and stationery support. Other capital investments made by the model (basic training, workshop, facility renovation), have been in place for quite a long time in MOHFW service delivery system but are not effectively meeting the needs of quality improvement of RH services. The DBRHC model has put forward evidence-based information for required low-cost modifications of the system. It is understandable that cost adjustment in basic and refresher training is possible through engaging the Technical Training Unit (TTU) of the Directorate General of Health Services (DGHS) and National Institute of Population Research and Training (NIPORT). For construction and renovation of health facilities the MOHFW has its own Health Engineering Department that can undertake suggested renovations. The MOHFW has already invested in maternal voucher scheme—where the model has suggested low-cost modified approach only. The expenses incurred for local travel by intervention staff and participants would be much lesser for MOHFW staff as they do not need to test the interventions at the field level. Given the above-mentioned resource mobilization using existing intra and interdepartmental functionaries for integrating the DBRHC model, the government sector has adequate scope to replicate the model through cost adjustment and perhaps at a lesser cost than estimated here. Similarly, NGOs working in reproductive health and family planning in Bangladesh have their own low-cost BCC material production arrangement, training/orientation units and funds for physical maintenance which can cope with the estimated cost of the DBRHC model through in-house cost adjustment.

## Conclusions

The built-in interventions of DBRHC model were doable in selected Government-and NGO settings at the grass-root level at a unit cost of US$3.38 per beneficiary. The model has potential to further reduce the cost if adjusted with the existing resources. Given the appropriate cost adjustment and resource mobilization by using available technical functionaries of the the Government and NGOs, cost of integrating the model is affordable.
